# Giant Cell Tumour of the Small Bones of Hand and Foot

**DOI:** 10.7759/cureus.42197

**Published:** 2023-07-20

**Authors:** Rahul Patel, Rahul Parmar, Srishty Agarwal

**Affiliations:** 1 Orthopedics and Traumatology, New Civil Hospital, Surat, IND; 2 General Medicine, New Civil Hospital, Surat, IND

**Keywords:** neo-adjuvant, surgery, bone tumor, small bones, giant cell tumor

## Abstract

Introduction

Giant cell tumor (GCT) or bony tumor mainly involving long bones of arms and legs is very rarely associated with the small bones of hands and feet. Due to its nonspecific signs and symptoms, it is not easy to diagnose based on clinical findings; therefore, histopathological evidence is required to confirm it.

Method

A total of 16 patients with positive histopathological bone lesions enriched with giant cells were included in our study. After a complete evaluation of their case records, the required radiological assessment was carried out. Campanacci's method of staging was used to evaluate the advancement of lesions. The Musculoskeletal Tumour Society (MSTS) score was recorded postoperatively. All the patients were followed up for a mean duration of 2.8 years until they were lost to follow-up.

Result

The result of the current study shows that 62.5% of our patients presented in their twenties and 81.25% of patients came at a reasonably advanced stage. Hand and foot were involved in 1:1 cases. Patients were treated by one of the following options: extended curettage with bone graft or cement, wide excision, or en bloc resection. Phenol, a neoadjuvant, was used in all patients. Two of our patients (6.25%) who underwent curettage with bone graft showed up with recurrence during follow-up - one was then treated with wide excision and the other with amputation.

Conclusion

Giant cell tumors should undoubtedly be aggressively approached with the goal of preserving limb function while reducing recurrence risk to as minimal as possible. GCT of hand is more aggressive comparatively and should be treated accordingly.

## Introduction

Giant cell tumor (GCT) is an aggressive benign tumor that is generally encountered in the meta-epiphyseal region of long bones. The knee and wrist constitute the most common site. Small bone is a term coined for a bone that lies distal to the radius and ulna in the hand and the tibia and fibula in the foot. Due to the varying availability of surgical options and the possibility of revision surgical risk (due to high recurrence rate), GCT has always been the center of controversy among surgeons, especially when it involves small bones of hand and foot, where functional outcome preservation should not outweigh the risk of associated metastasis. GCT of hand and foot occur in 1.7% to 5% of cases, respectively [1]. Many instances of their involvement in small bones have been reported; however, very few case series have been published.

The purpose of the current retrospective study was to determine the prognostic factors and recurrence risk related to aggressive surgical procedures for the management of GCT.

## Materials and methods

From a period of 2018-2022, 16 patients were identified who fit the criteria of having a GCT in small bones of the hand and foot. Small bone is a term coined for a bone that lies distal to the radius and ulna in the hand and the tibia and fibula in the foot. After institutional review board approval, a retrospective review of their medical details was conducted. Age at presentation, sex, symptoms, disease location, history of presentation, clinical, radiological, and pathological findings, as well as operative techniques and outcomes, were reviewed in full detail. Campanacci staging system was used to classify the lesions. All 16 patients were histopathologically confirmed to suffer from GCT of bone and were then included in the study. The Musculoskeletal Tumour Society (MSTS) score was recorded post-operatively. On radiological evaluation, one patient (6.25%) had pulmonary metastasis. Other patients did not show any other radiological sign of multicentric or metastatic GCT.

Two patients (12.5%) were operated on previously in another institution for GCT, one at the proximal phalanx of the middle finger and the other at the calcaneum, about six months ago. They presented us with a local recurrence of GCT at previously operated sites.

All the patients were followed up for a mean duration of 2.8 years (6-48 months).

## Results

**Figure 1:**
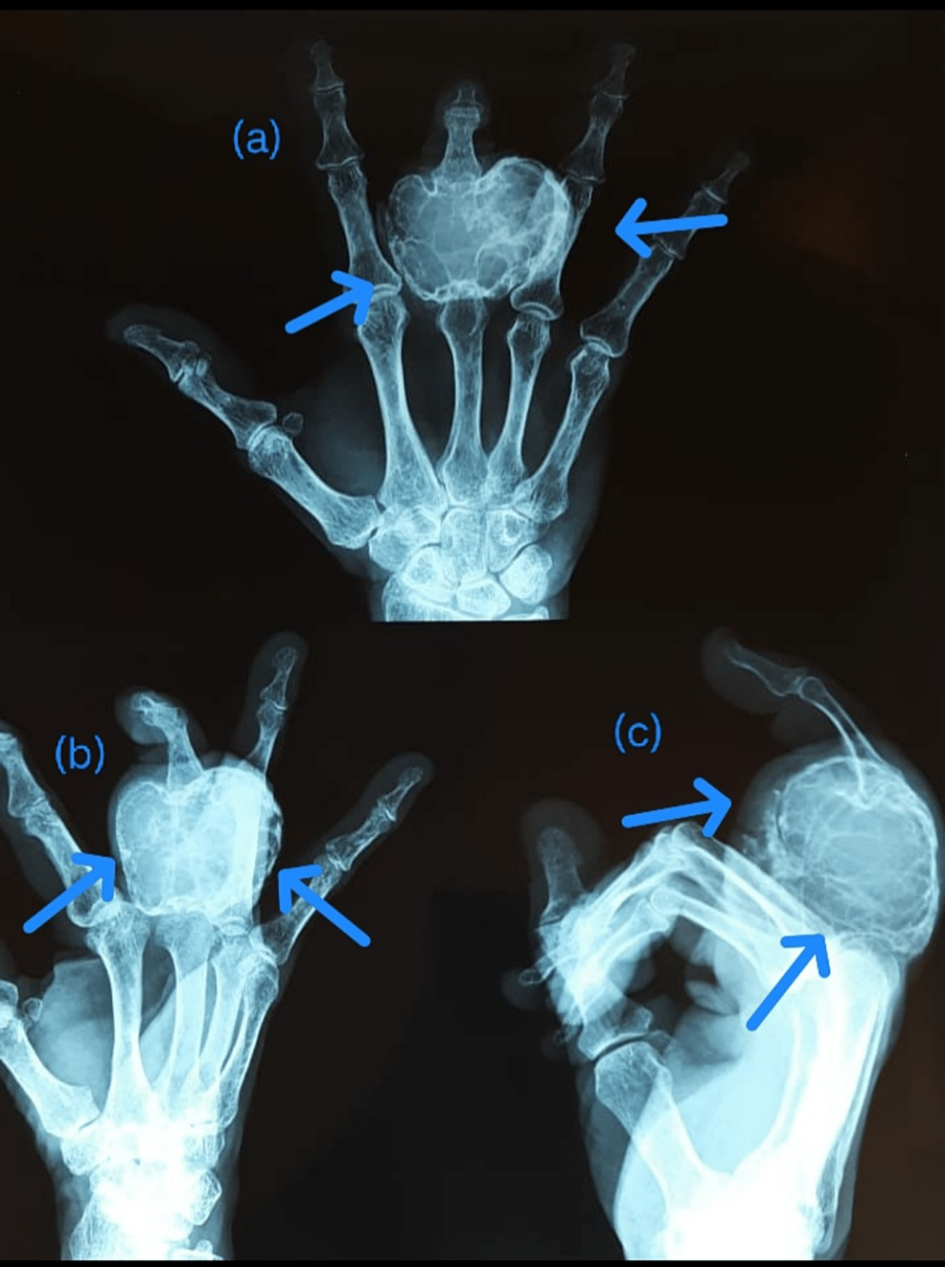
Case 2: Giant cell tumor (GCT) proximal phalanx of the middle finger A 50-year male patient had a complaint of swelling and pain over the middle finger proximal phalanx. After biopsy and confirmation of GCT, it was amputated.

**Figure 2:**
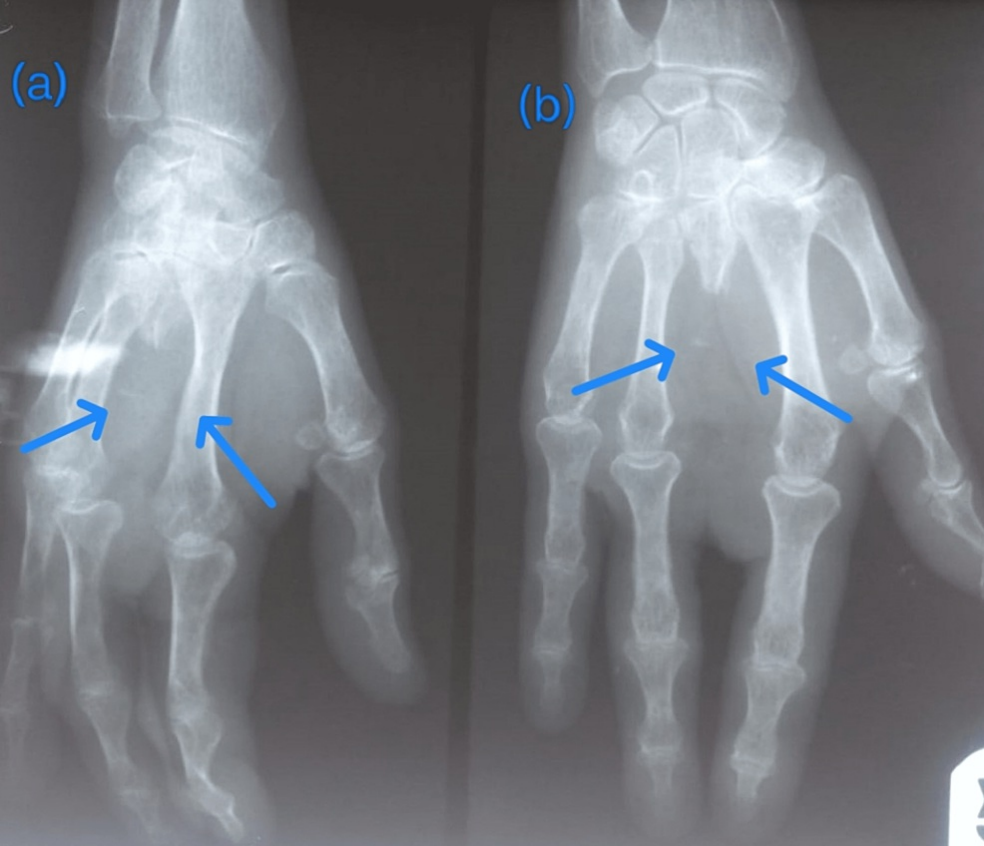
Case 2: postoperative X-ray

**Figure 3:**
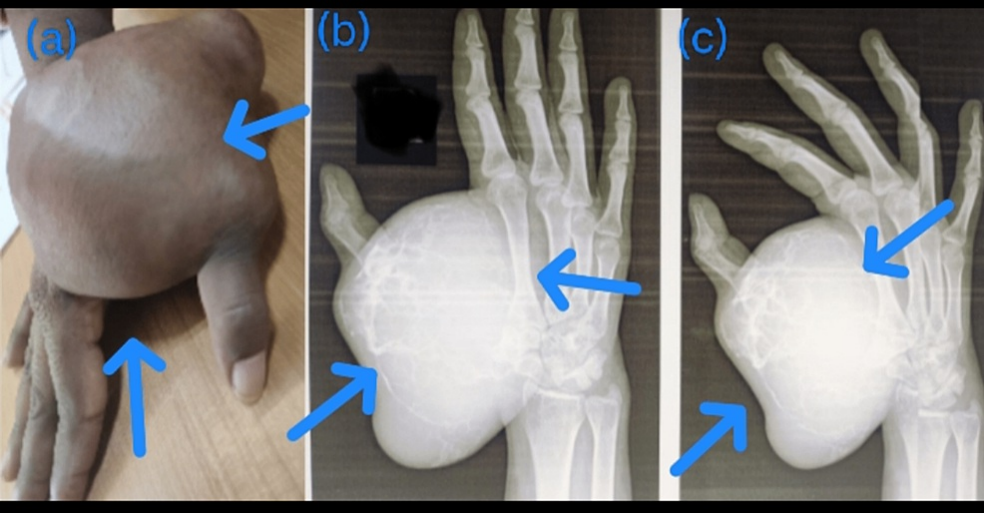
Case 1: fist metacarpal giant cell tumor (GCT) A 24-year-old male presented with an advanced-stage disease of the first metacarpal bone GCT with lung metastasis for which carpometacarpal disarticulation was done.

**Figure 4:**
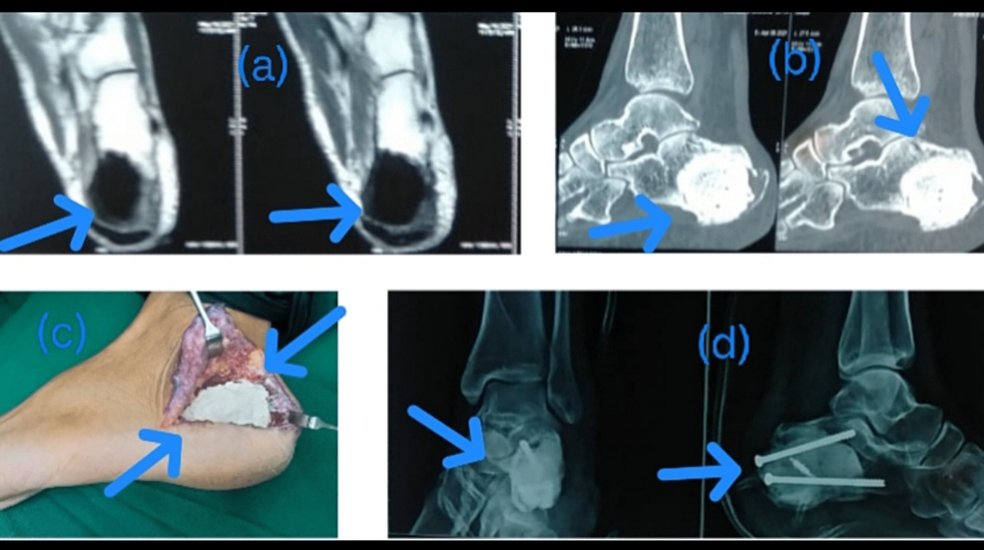
Calcaneum giant cell tumor (GCT) A 24-year-old male patient of GCT presented with a complaint of heel pain. After MRI (a) and CT scan (b) evaluation, and confirmation, it was treated with curettage (c) and cementing (d).

Of the 16 patients, eight patients (50%) had involvement in their hands, while the remaining eight (50%) had their feet involved (Table 1). Among those, the phalanx was involved in five patients (37.5%) (Figures 1, 2), and the metacarpal was involved in three patients (18.75%) (Figure 3). Out of the 50% of patients with foot involvement, talus was involved in three patients (18.75%); calcaneum in two patients (12.5%) (Figure 4); cuboid in one (6.25%); and metatarsal bones in the remaining two patients (12.5%) (Table 2). The mean age of patients was 34 years, ranging from 19-year-old female to 50-year-old male. Maximum patients (62.5%) presented to the clinic in their twenties. The male-to-female ratio was 1:1. Most patients presented with complaints of pain, which reduced postoperatively. On the radiological evaluation of the lesion, seven patients (43.75%) were classified in stage III, six patients (37.5%) were in stage II, and three patients (18.75%) were grouped in stage I according to Campanacci staging.

**Table 1:** Surgical techniques used in giant cell tumor of the small bone

Procedure	Hand	Foot
Curettage with bone graft	2	1
Curettage with cement	0	5
Wide excision	3	1
En bloc / Amputation	3	1

**Table 2:** Case series and treatment CMC: carpometacarpal

Sr.No.	Age	Sex	Site	Surgery/ Procedure
1	19	F	Proximal Phalanx, Middle Finger	Curettage+ Bone Graft
2	50	M	Proximal Phalanx, Middle Finger	Ray Amputation
3	45	M	4th Metacarpal	Wide excision+ Tricortical Iliac Bone graft
4	23	F	Talus	Curettage+ Bone Graft
5	48	F	1st Metatarsal	Ray Amputation
6	28	F	Middle Phalanx, Middle Finger	Wide Excision
7	24	F	1st Metacarpal	CMC Disarticulation
8	24	M	Talus	Curettage+ Cement
9	35	F	Calcaneum	Curettage+ Cement
10	24	M	1st Metatarsal	Wide excision+ Fibula Bone Graft
11	35	M	Proximal Phalanx, Ring Finger	Wide Excision+ Tricortical Iliac Bone graft
12	24	M	Calcaneum	Curettage+ Cement
13	23	F	Cuboid	Curettage+ Cement
14	25	M	Talus	Curettage+ cement
15	22	F	Proximal phalanx, Little finger	Ray Amputation
16	20	M	2nd metacarpal	Curettage+ Bone graft

Eight patients (50%) were treated with extended curettage and the use of phenol. The void was filled with either the use of bone graft in three patients (18.75%) or with cement in five patients (31.25%). Wide excision was performed in four patients (25%). The remaining four patients (25%) underwent en-bloc resection (Figures 3, 4). Two patients (12.5%) who were previously operated on outside our institution and presented to us with recurrence were treated with extended curettage and phenol with bone cementing. Phenol was used as a neoadjuvant in all our patients.

Patients were followed up until they were discharged from the clinic, died, or follow-up was irreversibly lost. Upon follow-up, we found that patients were relieved of pain and were satisfied. Two patients of GCT of hand (12.5%) showed recurrence within six months of their follow-up. They had previously undergone extended curettage with bone graft. During revision surgery, one of these patients underwent wide excision, while the other had to get an amputation done.

## Discussion

Giant cell tumor (GCT) is a primary bone tumor, 85% of which is found in long bones of the body and 10% in the axial skeleton, while the small bones of the hand and foot are found to be rarely involved [1]. The incidence of GCT is higher in Asian populations, with the highest reported incidence being in South India (30.3%) [2]. Most GCTs are present between the age of 30 to 50 years. However, GCT of small bones presents earlier, mainly in the twenties, which is observed by different studies on small bones affected by GCT, ranging from 28.5% to 45% [3,4,5]. In the current study, 10 out of 16 patients (62.5%) belonged to the age group of 20 to 30 years. The clinical significance of this fact still remains unclear.

Giant cell tumor of small bones is asymptomatic during the early stages. Owing to its multidirectional expansion, the tumor involves the cortex and surrounding soft tissues rapidly, thereby presenting in late stages. A total of 81.25% of our patients presented in stages II and III. In a study of 18 subjects conducted by Rajani et al. [6] on GCT of the foot and ankle, similar findings were concluded. Only 10% of their patients presented in stage I. On account of its late-stage presentation, major bony destruction and diaphyseal, as well as surrounding tissue extension, are seen, which limits its surgical options. Moreover, reconstruction after resection poses additional challenges in multicentric GCT. Luckily, none of our patients presented with multicentric GCT.

Recurrence of GCT in small bones has always bothered the operating surgeon, besides the patient. In the preceding studies, the recurrence rate of GCT in small bones has ranged from 25% to 50% [1, 3, 5, 6]. Biscaglia et al. [5] studied 29 cases of GCT of hand and foot and found a recurrence rate of 30% in his patients. Furthermore, the authors concluded that hands and feet appeared to behave aggressively compared to other bony GCTs. In accordance with other similar studies, GCT in the small bones of the foot was found to be less aggressive in comparison to other bony lesions of GCT. [7, 8]

Multiple studies conducted on GCT of small bones suggest that the involvement of the hand is very aggressive [3, 9, 10]. A study done by Yanagisawa et al. [3] on 11 patients found recurrence in two patients (18%) and metastasis in one patient (9%). These three patients (27%) had primary tumors located in the small bones of the hand. Keeping all this in mind, we implemented all the measures to prevent a recurrence, especially the lesions of the hand were managed promptly. Yet we found 12.5%, i.e., two cases of recurrence, wherein the primary site of the tumor was in the metacarpal. This supports the previously mentioned findings.

Different surgical modalities are available for treating GCT of small bones, and they all have varying functional outcomes and recurrence rates. Both the cases of recurrence that we faced were treated with extended curettage and bone graft. Averill et al. [10] conducted a multi-institutional study on 21 patients and 28 lesions. The local recurrence amounted to 90% with curettage or curettage and bone grafting. In another study by Athanasian et al. [11] on 14 patients, 79% accounted for recurrence whose primary lesion was treated with curettage. However, these studies lacked the use of any neoadjuvant. The current study used phenol as an adjuvant, yet, two patients presented with recurrence, which could be the reason behind the lower recurrence rate. Rajani et al. [6] also advocated the use of neoadjuvants in their study and recommended not to use curettage or bone grafting alone. Unfortunately, the current study is limited by the retrospective method and a smaller number of cases due to the sporadic nature of GCT in small bones of the foot and hand.

Various studies have advocated the use of different agents as adjuvants. However, due to cost-efficiency in a country like India, the use of such agents is currently very limited. Apart from phenol, hydrogen peroxide is also readily available and cost-effective and thus can be used as an efficient neoadjuvant. Omlor et al. [12] commented on the efficacy of hydrogen peroxide use locally to clean the cavity and drew the inference that hydrogen peroxide increased the recurrence-free survival rate. Tse et al. [13] studied the effect of oral bisphosphonate on GCT bone lesions and found a reduction in the recurrence rate of lesions in patients treated with oral bisphosphonate in comparison to patients who did not receive the oral treatment. Nevertheless, further studies and trials must be carried out to evaluate their effect on the GCT of small bones.

## Conclusions

GCT of small bones is a primary bone tumor that affects the young age group (the 20s) and presents at advanced stages. GCT of hand is more aggressive and is associated with high recurrence rates. The use of neoadjuvant does not eliminate the risk of recurrence but possibly reduces its rates. Surgeons must strike a balance between the functional outcome of the chosen method and the risk of its recurrence while operating on these fairly young patients.
